# Multifocal multiphoton volumetric imaging approach for high-speed time-resolved Förster resonance energy transfer imaging *in vivo*

**DOI:** 10.1364/OL.43.006057

**Published:** 2018-12-12

**Authors:** Simon P. Poland, Grace K. Chan, James A. Levitt, Nikola Krstajić, Ahmet T. Erdogan, Robert K. Henderson, Maddy Parsons, Simon M. Ameer-Beg

**Affiliations:** 1 Comprehensive Cancer Centre, School of Cancer and Pharmaceutical Sciences, Guy's Campus, King's College London, UK; 2 Randall Centre for Cell and Molecular Biophysics, Guy’s Campus, Kings College, UK; 3 Institute for Integrated Micro and Nano Systems, School of Engineering, University of Edinburgh, Edinburgh, UK; 4 EPSRC IRC “Hub” in Optical Molecular Sensing & Imaging, Centre for Inflammation Research, Queen’s Medical Research Institute, 47 Little France Crescent, University of Edinburgh, Edinburgh, UK

## Abstract

In this Letter, we will discuss the development of a multifocal multiphoton fluorescent lifetime imaging system where four individual fluorescent intensity and lifetime planes are acquired simultaneously, allowing us to obtain volumetric data without the need for sequential scanning at different axial depths. Using a phase-only spatial light modulator (SLM) with an appropriate algorithm to generate a holographic pattern, we project a beamlet array within a sample volume of a size, which can be preprogrammed by the user. We demonstrate the capabilities of the system to image live-cell interactions. While only four planes are shown, this technique can be rescaled to a large number of focal planes, enabling full 3D acquisition and reconstruction.

Due to its relative independence to absolute intensity value, fluorescence lifetime imaging (FLIM) can overcome issues associated with steady-state fluorescent techniques. The average lifetime of a fluorophore varies according to its local environment and has been used to measure Förster resonance energy transfer (FRET) [1,2], pH [3], protein binding [4] (i.e., NADH), relative ion concentrations, local variations in viscosity [5], aggregation [6], and proximity to metal surfaces [7].

For high-precision FLIM, time-correlated single-photon counting (TCSPC) is unparalleled in its measurement accuracy. Conventional TCSPC is fundamentally limited with respect to the photon counting rate in current implementations of laser scanning microscopy, with typical acquisition rates for conventional laser scanning TCSPC FLIM on the order of minutes. This partly explains why its application is not more widespread in the biomedical community. Until recently, high-speed FLIM could only be performed using modulated or time-gated image intensifier systems [8–10]. While such systems offer video frame rate acquisitions, they suffer from significant imaging artefacts [11] and excitation photon flux that may be damaging to cells [12–15].

We have previously presented a massively parallel, fully addressable time-resolved multifocal multiphoton microscope [16,17] capable of producing fluorescence lifetime images with 55 ps time-resolution giving improvements in acquisition speed of a factor of 64, improving the acquisition time to the order of seconds. Parallelized TCSPC detection was achieved using a specialized 32×32 10 bit time-to-digital (TDC) array (∼55ps) with integrated low dark-count single-photon avalanche photodiodes (SPAD) [18].

The concept of acquiring several axially separated focal planes simultaneously in multiphoton microscopy is not new and has already been presented by a number of groups utilizing techniques such as remote focusing [19,20], spectral encoding [21], and holographic approaches [22]. The benefits of acquiring several planes simultaneously over acquiring a single plane (albeit at high speed) are that volumetric data are acquired without the need for axial translation of the objective or sample. This axial translation is still limited by speed and can also cause perturbation to the sample.

In this Letter, we will discuss a modification of a multifocal multiphoton system (MM-FLIM), which enables simultaneous acquisition of four individual planes of both fluorescent intensity and lifetime information, allowing us to obtain volumetric data without the need for sequential scanning at different axial depths.

MM-FLIM setup and general operation have been described in much greater detail elsewhere [16,17]. In brief, light generated from the Chameleon Ultra II laser (Coherent) is projected onto an HSPDM512 spatial light modulator (SLM) device (Meadowlark Inc.). By applying a suitable phase pattern, a two-dimensional array of 8×8 beamlets is produced, which is then raster scanned using a set of galvanometer scanners and projected onto the sample. The fluorescence generated from each beamlet is collected, descanned, and projected onto the Megaframe camera (Photon Force Ltd.). Each beamlet is precisely aligned to its associated detector matching the spacing and angular orientation of the array to enable high collection efficiency. For multiphoton excitation, fluorescence is only generated within the focal volume where photon density is sufficiently high. When optically conjugate, the fluorescent beamlet projected onto the detector aperture is significantly smaller (1.8 μm FWHM) than the active area of the SPAD (6 μm dia.) due to the choice of the reimaging objective (Nikon 10×, 0.3 NA). It should be noted that one could apply a small defocus term to the excitation beamlet and still collect the light from the focal volume even if the detector is not completely conjugate to focal point. Due to the small size of the detector, it will only be effective within a certain z-range. From each beamlet, a subimage is generated from a raster scan, which can be then stitched together to create a complete image. All sample imaging is performed using a 40×1.3 N.A. Plan Fluor oil immersion microscope objective (Nikon Instruments Ltd.)

The generation of uniform beamlet arrays in a single plane using a doubly weighted Gerchberg–Saxton algorithm (DWGS) in conjunction with a SLM has been described previously [23]. We use a modified version of this algorithm to generate the desired uniform 3D distribution of diffraction-limited spots (Fig. 1). A known defocus term is applied to each individual beamlet corresponding to its relative plane position with the four planes centered axially about a zero z-offset position. In the first iteration of the modified DWGS algorithm, the randomly generated phase pattern and the premeasured laser illumination amplitude are coupled, forming a complex incident field, and a Fourier transform is made to determine the amplitude (V) and phase (φ) components at the image plane. The simulated beamlet pattern is compared with the desired beamlet array, taking the z-offsets of each beamlet into account, and a suitable weighting is applied to produce a new amplitude. This is combined with the phase, which was previously generated at the image plane, and once the inverse Fourier transform is carried out, the first iteration of the algorithm is completed. The phase pattern generated in the previous iteration is then fed back into the next iteration, where it is coupled with the measured laser amplitude, and the process is repeated. After 30 iterations of simulated beamlet generation and feedback, the calculated phase is projected onto the SLM, and the associated fluorescent beamlet array signal feedback generated from a homogeneous fluorescent sample is detected by the SPAD array. These beamlet array signals are then normalized for the quadratic effect of two-photon excitation. The inverse response is then determined and incorporated as the new desired beamlet output. This process is repeated a number of times until the beamlet uniformity is maximized.

**Fig. 1.**
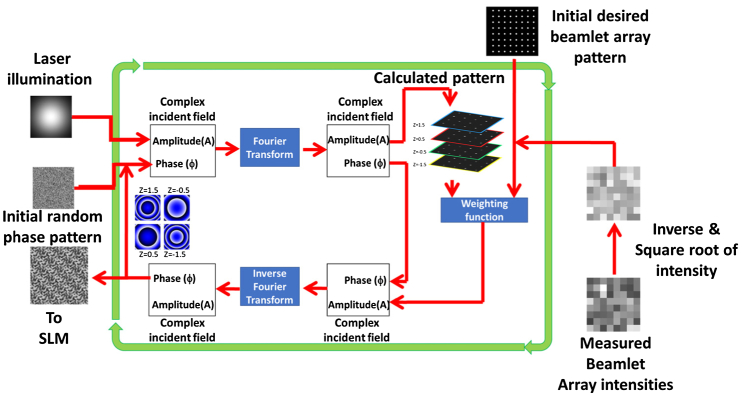
Operation of the modified DWGS algorithm.

For an 8×8 array, in order to generate four sequential axial planes of equidistant spacing (z), where each plane significantly overlaps spatially in x and y, a particular z-offset pattern was applied to the beamlet array [see Fig. 2(a)]. Each individual z plane consists of 4×4 beamlets, and as each adjacent beamlet corresponds to a different plane, the beamlets must be raster scanned over twice the distance required in x and y for a single beamlet in a single plane 8×8 acquisition in order to generate a complete image. The generated image is composed of four planes, each containing 16 subimages, as shown in Fig. 2(b).

**Fig. 2.**
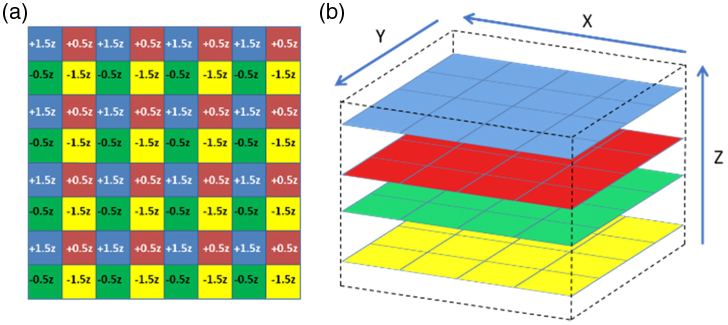
(a) Z offset map showing the applied z for each beamlet in the 8×8 array and (b) their orientation in space.

In order to test the ability of the system to vary the interplanar z-offset and acquire data sets at multiple planes simultaneously, we measured the fluorescence axial response with an autofluorescent diagnostic slide (Chroma Inc.) with 870 nm excitation. The diagnostic slide provides a homogeneous fluorescent signal and effectively acts as a fluorescent sea. By calculating the differential of this response, one can determine the surface position from the peak. In Fig. 3, the differential of this axial response for each plane is presented with interplanar spacings (z) of 0.5, 1.0, 1.5, and 2.0 μm.

**Fig. 3.**
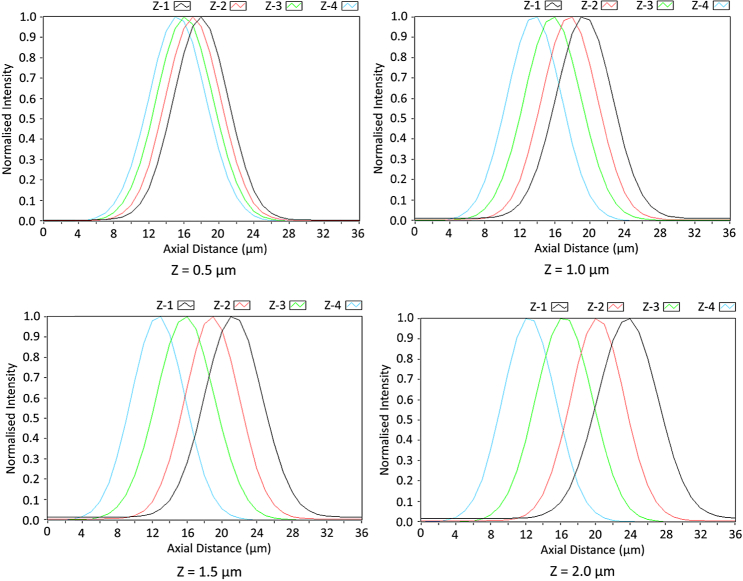
Differential of the axial response of the system at interplanar spacings of 0.5, 1.0, 1.5, and 2.0 μm.

To generate complete images in each of the four planes, each beamlet must be overscanned by a factor of 2 in both the x and y axes as each adjacent beamlet is allocated to another plane. The user simply applies the precalculated phase pattern with the appropriate interplanar spacing required and acquires an image composed of 8×8 subimages, which are then processed to give 4×4 subimages for each plane, as shown in Fig. 4.

**Fig. 4.**
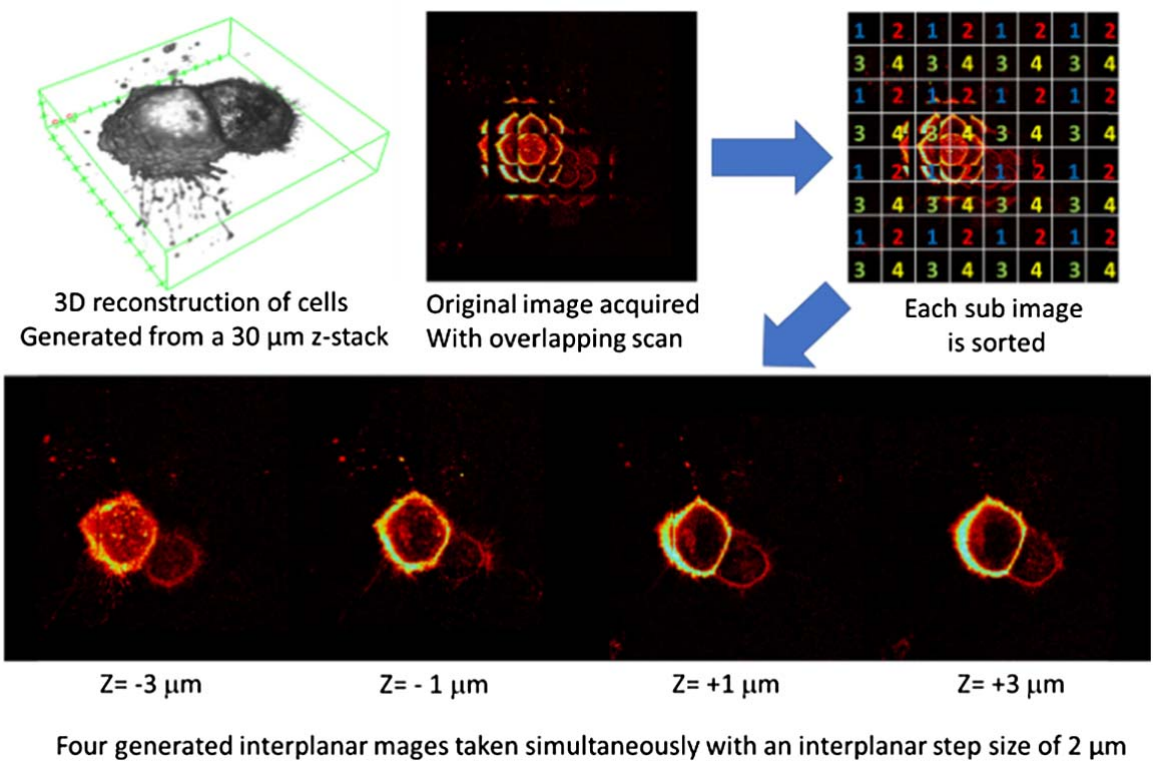
Sorting procedure used to organize four planar data sets (top) into their constituent frames (bottom).

As seen in Fig. 4, the 3D image is first constructed from a 30-image single-plane z-stack. From this data set, the central z position is chosen, and the precalculated four-planar beamlet array pattern with z offset (in this case 2 μm) is projected onto the SLM. A raster scan is performed for each beamlet array, and the subimages from the original image acquired are sorted into their associated planes, denoted by their corresponding number.

To demonstrate the dynamic imaging capability of the system, we imaged live human epithelial cells expressing a RhoA GTPase mTFP/Venus FRET biosensor [24]. Images were acquired at 0.1 Hz before and after media was exchanged for ${\mathrm{Ca}}^{2+}$-free media to induce cell–cell dissociation through disengagement of cadherin receptors (Fig. 5).

**Fig. 5.**
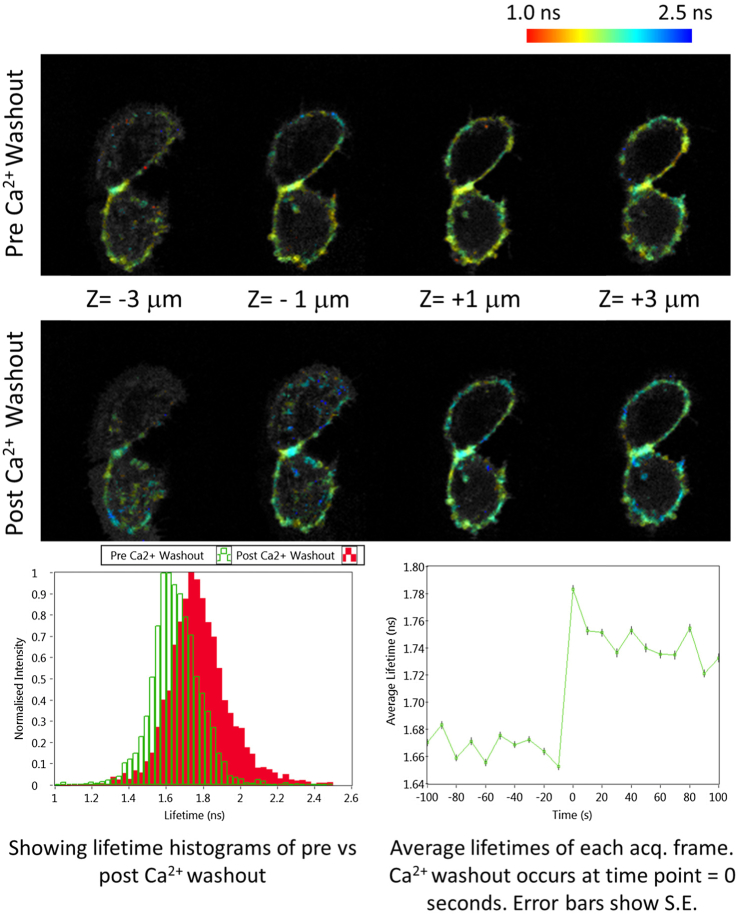
Presenting volumetric intensity-weighted FLIM data of epithelial cells expressing mTFP/VenusRhoA GTPase FRET biosensor pre- and post-${\mathrm{Ca}}^{2+}$ washout to induce junction disassembly.

Analysis of spatial changes in RhoA activation revealed a significant increase in fluorescence lifetime at cell–cell junctions from 1.65±0.14 to 1.78±0.18ns following removal of ${\mathrm{Ca}}^{2+}$, shown at time point 0 s in Fig. 5, consistent with a reduction in active RhoA and resulting actomyosin contractility as previously suggested [25,26]. The control lifetime of mTFP was measured at 2.15 ns, indicating that resting cell GTPase activity of the biosensor corresponds to a FRET efficiency of 23%, which is consistent with Fritz *et al.* [24]. The 3D projection enables us to interrogate the architecture of the cell and has the potential to provide unprecedented spatiotemporal resolution regarding RhoA activity within cells relative to their 3D position.

While this is only a proof of principle with a four-plane acquisition, with more beamlets, this technique can be rescaled to a large number of focal planes, enabling full 3D acquisition and reconstruction. In the future, the generation of 32×32 beamlets will allow 8×8 beamlet acquisition of 16 individual planes simultaneously. At present, we are limited to the number of beamlets we can generate due to the laser power requirements for multiphoton excitation and the low optical efficiency of the SLM used (∼25%). Moving to a custom-designed diffractive optical element with high optical efficiency would enable full utilization of the Megaframe camera.
